# Strategies to Improve the Clinical Utility of Saporin-Based Targeted Toxins

**DOI:** 10.3390/toxins10020082

**Published:** 2018-02-13

**Authors:** Francesco Giansanti, David J. Flavell, Francesco Angelucci, Maria Serena Fabbrini, Rodolfo Ippoliti

**Affiliations:** 1 Department of Life, Health and Environmental Sciences, University of L’Aquila, I-67100 L’Aquila, Italy; francesco.giansanti@cc.univaq.it (F.G.); francesco.angelucci@univaq.it (F.A.); 2 The Simon Flavell Leukaemia Research Laboratory (Leukaemia Busters), Southampton General Hospital, Southampton, SO16 8AT, UK; davidf@leukaemiabusters.org.uk; 3 Italian Ministry of Education, I-20100 Milano, Italy; msfabbrini@gmail.com

**Keywords:** *Saponaria officinalis*, immunotherapy, ribosome-inactivating proteins, monoclonal antibodies, anti-cancer therapy, chimeric toxin

## Abstract

Plant Ribosome-inactivating proteins (RIPs) including the type I RIP Saporin have been used for the construction of Immunotoxins (ITxs) obtained via chemical conjugation of the toxic domain to whole antibodies or by generating genetic fusions to antibody fragments/targeting domains able to direct the chimeric toxin against a desired sub-population of cancer cells. The high enzymatic activity, stability and resistance to conjugation procedures and especially the possibility to express recombinant fusions in yeast, make Saporin a well-suited tool for anti-cancer therapy approaches. Previous clinical work on RIPs-based Immunotoxins (including Saporin) has shown that several critical issues must be taken into deeper consideration to fully exploit their therapeutic potential. This review focuses on possible combinatorial strategies (chemical and genetic) to augment Saporin-targeted toxin efficacy. Combinatorial approaches may facilitate RIP escape into the cytosolic compartment (where target ribosomes are), while genetic manipulations may minimize potential adverse effects such as vascular-leak syndrome or may identify T/B cell epitopes in order to decrease the immunogenicity following similar strategies as those used in the case of bacterial toxins such as Pseudomonas Exotoxin A or as for Type I RIP Bouganin. This review will further focus on strategies to improve recombinant production of Saporin-based chimeric toxins.

## 1. Introduction

Since the idea of the “magic bullet” was conceived by Paul Erlich [1] over one hundred years ago, there has been a competitive surge to determine the best toxic molecule to deliver into cancer cells. The ideal molecule must avoid cellular resistance and be capable of repeated cytotoxic action against the target cells. In this context, an enzyme endowed with the characteristics matching these requirements is Saporin produced by the common soapwort plant *Saponaria officinalis*. It was firstly described by the group of Fiorenzo Stirpe [2], and appeared as one of the most appealing candidates as a constituent of the magic bullets. Recently, Saporin (the main isoform is SO6 from seeds, also named Sap-SO6) has been largely investigated as antitumor agent in several clinical trials but with mixed success, due to toxicity problems also common to other ribosome inactivating proteins (RIPs) that have substantially hampered their employment in a clinical setting. This review examines the available data on RIPs with particular attention to Saporin and on their conjugates with antibodies (immunotoxins, ITx) and chimeras (i.e., fusion proteins containing RIPs as the toxic moieties) suggesting new strategies to improve their efficacy in order to open new routes for the development of next generation anti-cancer agents.

## 2. Saporin Structure and Function

Saporin is a toxic protein produced by *Saponaria officinalis* L., a member of the Caryophyllacea family that is classified as a Type I ribosome-inactivating protein (RIP). This plant produces several isoforms of Saporin, the major one being SO6 from seeds, also named Sap-SO6. Mixtures of closely related isoforms and several cDNA and genomic clones have been isolated from *Saponaria officinalis* L. Sap-SO6 represents the major HPLC peak (peak 6) of purified seed-extracted protein and constitutes up to the 10% of the total protein content Seed protein sequencing revealed heterogeneity at two positions, showing either an aspartic or a glutamic acid in position 48, and either lysine or arginine present in position 91, thus indicating that the SO6 peak contains a set of closely related Saporin isoforms. Single recombinant expression of these seed isoforms demonstrated they had same catalytic activity, except for a leaf-derived cDNA that corresponded to a previously identified SAP-5 isoform that showed a 10-times lower catalytic activity [3]. We and others have used the seed extracted SO6 peak for ITx chemical construction or one of the seed isoforms (SAP-3) for the recombinant expression of chimeric toxins (see below).

Saporin is comprised of a single catalytic chain that is devoid of any lectin-like binding domain. This is in contrast to ricin holotoxin that is comprised of two protein chains, the A chain possessing N-glycosidase catalytic activity and the B chain possessing lectin binding activity. The two chains are linked together by a single disulfide bond whose reduction is necessary to allow the free A chain [4,5] damaging ribosomes within the cytosol of a cell, once internalized.

Saporin and all plant RIPs possess N-glycosidase activity (EC 3.2.2.22), which removes a specific adenine (A4324 in the 28S ribosomal rRNA) located in a universally conserved GAGA-tetra loop also termed the α-sarcin/ricin loop. This specific depurination leads to a permanent inactivation of the large ribosomal subunit, blocking the recognition and binding of the elongation factor EF-1 and the subsequent formation of the EF-2/GTP-ribosome complex thereby preventing the translocation of the tRNA from the A site to the P site [6] of the ribosome complex, thus irreversibly blocking protein synthesis. Consequently all RIPs are cytotoxic for mammalian cells, with LD50’s in mice varying from few milligrams/kg for Type I RIPs to few micrograms or less for Type II RIPs as Ricin [7].

Type I RIPs have high isoelectric points (pI > 9.5), while on the contrary Type II catalytic A chains are either neutral (pI 7.09 for RTA) or acidic (pI 5.25 for Abrin A chain) [8]. Despite these differences, the 3D-structures of these proteins are largely superimposable with the key catalytic residues being conserved among all toxins belonging to RIP family [9,10].

The mature form of Sap-SO6 consists of 253 amino acids almost ten percent of which is represented by lysine residues, a structural feature that confers an extremely high pI of approximately 10 [11].

**Figure 1:**
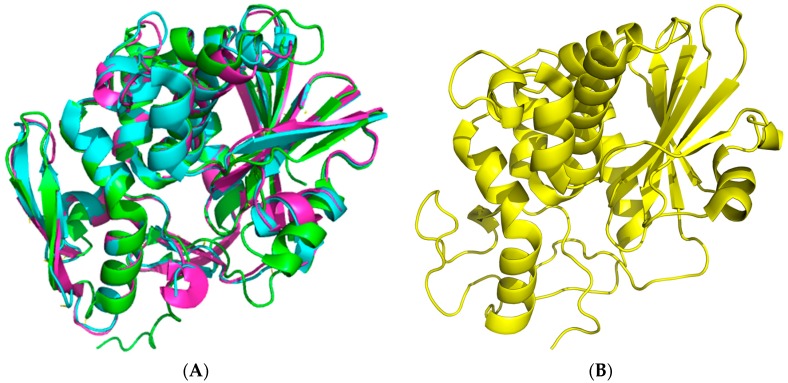
Cartoon 3D-representations of seed Saporin (Sap-SO6) and of its representative homologous RIPs using the program PyMol [12]. (**A**) The crystal structure at 2.0 Å of Sap-SO6 (PDB code: 1QI7) [9]; (**B**) Superposition of three representative RIPs Ricin Toxin A (RTA), Ricin A chain, PDB code: J1M (cyan); Momordin, PDB code: 1MOM (magenta); Thrichosanthin, PDB code: 1TCS (green) [13,14,15].

The crystal structure of seed-extracted Sap-SO6 (PDB Code: 1QI7) has revealed that this protein contains two main domains: an N-terminal domain with a predominantly beta-sheet structure and a C-terminal domain with a prevalent alpha-helix structure (Figure 1A). The N-terminal domain is very similar to that of the other RIPs and contains six β-strands arranged to form a β-sheet, four antiparallel sheets in the center and the other two at the edges that, instead, run parallel. The C-terminal domain contains eight α-helices. The helices A and B are part of the cross-connections between the parallel filaments of the β-sheet. The helices E and F are contiguous in sequence and a single residue (Phe180) assumes a non-helical conformation, introducing a loop between them [9]. This structural arrangement is conserved among all Type I RIPs and the A chain of Type II RIPs (see Figure 1B) from plants.

**Figure 2:**
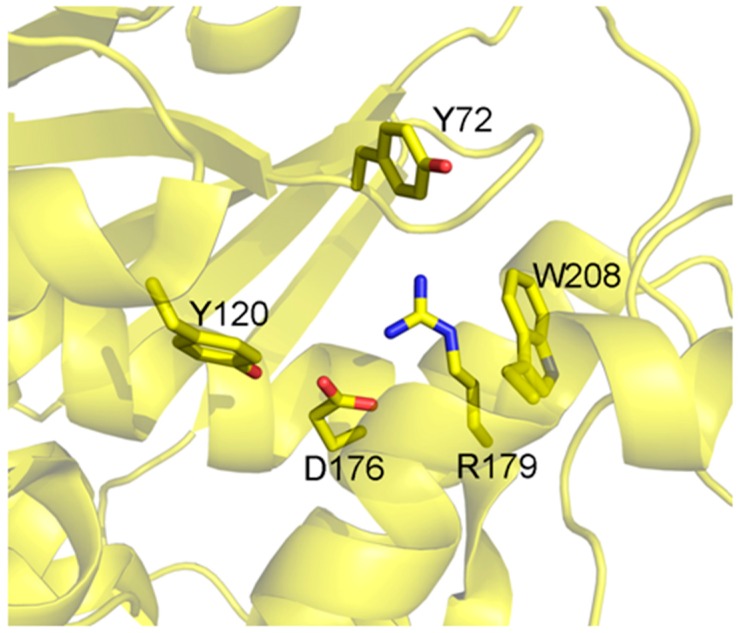
Catalytic site of Sap-SO6. Residues involved in the catalytic activity of Sap-SO6 are displayed in sticks [9].

The active site (Figure 2) is conserved between the different isoforms of Saporin, being composed by Tyr72, Tyr120, Glu176, Arg179 and Trp208 (residue numbers referred to are for the Sap-SO6 isoform) [16,17].

The key catalytic residues Glu176, Arg179 and Trp208 also take up the same position in other RIPs while Tyr72 assumes a different conformation, with this residue being responsible for the interaction with the target ribosomal adenine [9].

The Sap-SO6 isoform is extremely resistant to high temperatures, to denaturation with urea or guanidine or to the attack by proteolytic enzymes [18]. Furthermore, Sap-SO6 can also retain its catalytic activity in response to chemical modifications, such as those used for derivatization or chemical conjugation procedures [19]. All these peculiar characteristics made Sap-SO6 a promising candidate for the construction of immunoconjugates [16,20].

In order for Saporin to show therapeutic utility, the toxin must be able to gain entry into the cytosol of the target cells; it must therefore overcome the fundamental barrier presented by the plasma and intracellular membranes that constitute the endo-lysosomal system. Type II RIPs, due to the presence of a lectin moiety that binds to sugar residues on the plasma membrane surface, have an efficient route of access to the cell interior via endocytic processes, that subsequently lead them to a retrograde transport pathway trafficking through the Golgi complex to the ER [19]. On the contrary, Type I RIPs have a low cellular internalization efficiency [21], and generally follow intracellular pathways different from those of the prototypical Ricin AB toxin [16,22,23].

With regard to Saporin, the observations that some cell types show a moderate resistance to its cytotoxicity and that other cell types are much more sensitive, led some researchers to identify specific surface receptors that may mediate the internalization of this plant toxin. A mechanism of endocytosis mediated by the α-2-macroglobulin receptor [24] was proposed; despite there being a discrepancy between the levels of the receptor protein and the cytotoxicity induced by the Saporin. Some cell lines, positive or negative for expression of this receptor, showed a similar sensitivity to the effects of Sap-SO6 suggesting the possibility of an alternative endocytic mechanism independent of this receptor [25]. However, the rip Trichosanthin has also been shown to bind to the α-2-macroglobulin receptor or LRP, and several data have been produced independently to show these interactions [26].

## 3. Saporin as a Component of Immuno- or Chimeric Toxins

Saporin has been attached to a variety of different murine monoclonal antibodies to generate first-generation “immunotoxins” directed against several different cancer types, particularly hematological malignancies [20].

Pioneering work was done with chemically linked immunotoxins by the group of Stirpe in Bologna and Flavell in Southampton, directing Saporin to human leukemia and lymphoma cells expressing differentiation antigens such as CD2, CD7, CD19 and CD22 on their plasma membrane surface [27,28,29,30,31,32,33,34,35,36]. In all of these studies the immunotoxins were demonstrated to act at sub-nanomolar concentration in vitro while exerting significant in vivo therapeutic effects in animal xenograft models. The first reported clinical trial with a Saporin-based immunotoxin dates back to 1992 [37], when an anti CD30-Saporin was used to treat refractory Hodgkin lymphoma patients. The study first demonstrated a clinical efficacy in reducing the tumor mass by up to 60%. The therapeutic effect was limited to 2–4 months but clearly demonstrated the possibility to use Sap-SO6-based immunotoxins to treat hematological malignancies. In this preliminary study toxicity problems were encountered, due to side effects of Saporin, mainly caused by vascular leak syndrome (VLS) (see below) and the production of antibodies against both the toxin and the monoclonal antibody [38].

Moreover, a clear cellular cytotoxicity (ADCC) depending on the presence of the Fc portion of the antibody [20] suggested that the therapeutic potential may be limited by the use of antibody fragments as carriers for the toxin.

Saporin-based immunotoxins containing anti-CD22 bispecific antibodies were also tested in pilot clinical trials, with heterogeneous clinical effects, low signs of general toxicity, however, with some patients developing grade I VLS or myalgia, fever and development of anti-Sap-SO6 or anti-mAb antibodies [37,38,39]. 

A comprehensive list of Saporin-based immunotoxins reported in the literature has been recently published [20]. All have shown selective cytotoxicity for the appropriate antigen expressing cell line with IC_50_ values in the nano to pico molar range displaying differences from hundreds to thousands-fold increases compared with cells exposed to Saporin toxin alone.

### 3.1. In Vivo Performance of Saporin-Based Immunotoxins and Targeted Toxins in Preclinical Models

There is a considerable amount of data available on the in vivo performance of Saporin-based immunotoxins used individually or in combinations in preclinical xenograft models of human cancers in severe combined immunodeficient (SCID) mice. A selected number of these are presented in Table 1.

### 3.2. Clinical Experience with Saporin-Based Targeted Toxins

There is some limited clinical experience with Saporin-based immunotoxins and other types of targeted toxin and these are listed in Table 2. All of the reported clinical trials used the Saporin SO6 isoform. The earliest report is on the use of an anti-CD30-Saporin immunotoxin BER-H2/SO6 in patients with CD30^+^ Hodgkin’s lymphoma [40]. Only four patients were treated in this pilot study and three showed objective major responses without any attendant serious toxicity. French and coworkers [41,42] treated five low grade B-cell lymphoma patients with a mixture of two bispecific antibodies (BsAbs), both directed against CD22 but each with the second Fab arm directed against a different epitope on the Saporin molecule. The BsAbs were pre-complexed with the SO6 isoform of Saporin prior to administration to patients. Separate phase I clinical trials were undertaken with the anti-CD19 IT BU12-Saporin one in adult patients with relapsed/treatment resistant B-NHL and the other in pediatric patients with relapsed pre-B-cell acute lymphoblastic leukemia. In both trials the MTD (Maximum Tolerable Dose) was never reached, minimal toxicities were reported with two NHL patients in the first trial showing minor responses. Outcomes in the pediatric relapsed ALL trial were difficult to interpret because of the poor state of health of these patients and the trial was stopped prematurely for this reason. In a phase I trial with the anti-CD38 IT OKT10-Saporin in myeloma patients, minor responses were noted in two of the ten patients treated. Again, the MTD was never reached in this study, which was also closed prematurely because of concerns that off-target toxicities may occur following the availability of new mid-trial data on tissue expression of CD38. Human anti-Saporin antibodies were not detected in any of the treated patients though this may have reflected the immunocompromised state of the heavily pre-treated patient population involved in this study.

## 4. How to Solve the Problem of Immunogenicity of Plant Rips: Analysis of Experimental Data and Comparative Predictions for Saporin

It has been reported that approximately 40% of patients with a variety of hematological tumors respond to immunotoxins, produced using both bacterial or plant toxins, with the production of anti-toxin antibodies [47,48,49], but nonetheless may still undergo multiple treatments with immunotoxins [47]. In contrast 50–100% of patients with solid tumors produce neutralizing anti-toxin antibodies (50–100%) and cannot receive more than a single dose [48], thus greatly decreasing the therapeutic potential of these anticancer drugs. This discrepancy may be due to a greater accessibility of hematological tumors to immunotoxins combined with a more profound patient’s immunocompromised state in comparison to patients with solid tumors. Notwithstanding the possibility to treat some forms of solid tumors by local administration (i.e., neck and bladder [50,51]) thus reducing the exposure to the immune system, there is an urgent need to identify the most critical epitopes on RIPs so as to reduce their immunogenicity and thus improve their therapeutic utility. In this respect a number of RIPs have been investigated to identify the major antigenic determinants responsible for inducing an immune response. In this section, the conservation of residues identified in RIPs other than Saporin will be analyzed and predictions on Saporin immunogenic sites will be made.

Recently Cizeau et al. [52] described a mutagenesis approach to reduce immunogenicity of the plant RIP Bouganin. They identified a number of Bouganin peptides that induced T-cell proliferation and through in silico analysis identified the residues presented as possible antigenic determinants for MHC class II recognition. They expressed a Bouganin mutant (deBouganin) carrying four mutations (V123A, D127A, Y133N, I152A) that did not affect the catalytic activity of the enzyme but did significantly reduce the immune response of the host versus the toxin. At present, deBouganin-based immunotoxins are under investigation in a Phase I clinical trial sponsored by VIVENTIA Biotech Inc. (Winnipeg, MB, Canada) [53].

Pioneering work was undertaken by Mulot et al. [54] on the de-immunization of Trichosanthin, a well studied plant RIP, because of its anti-viral potential, particularly against HIV. They identified very large peptide segments (positions 1–72, 101–152 and 153–246) as potential antigenic sites. Due to the common use of Trichosanthin in traditional Chinese medicine, other groups have also investigated the problem of immunogenicity of this toxin. An et al. [55] characterized mutants of this RIP, where two potential antigenic sites (peptides YFF 81–83 and KR 173–174) were selected on the basis of a structural analysis. Mutants of Trichosanthin in these regions showed unaltered enzymatic activity and a reduced immunogenicity with respect to an induced IgE antibody response in immunized animals, but did not completely prevent an IgG antibody response. Gu et al. [56] identified at least four different epitopes on the Trichosanthin molecule by using a competitive antibody assay, while the group of Chan [57] achieved a reduction in immunogenicity but also in catalytic activity following the deletion of the last seven residues at C-terminus of the Trichosanthin molecule. Zhu et al. [58] predicted the presence of two immunogenic regions in peptides 21–27 and 41–48. Cai et al. [59] identified the region 169–174 partially overlapping with the region described by An et al. [55]. Chan and He [60,61] successfully mutated residues S7C, K173C and Q219C and added cysteine residues suitable for PEG or dextran modification that resulted in a substantial reduction in the immunogenicity of Trichosanthin [62]. Zhang et al. [63] finally identified two specific residues (Y55 and D78) exposed on the surface of the toxin that upon mutation (Y55G and D78S) strongly reduced the immunogenicity of this toxin in mice. It is evident from the published data for Trichosanthin, that there are a multiplicity of potential immunogenic sites on the molecule that to date have not yet been fully elucidated. 

Leung et al. [64] mapped three different epitopes on the surface of alpha-Momorcharin, another anti-viral and anti-cancer protein widely used in Chinese medicine. The three regions (1–14, 71–136 and 195–222) were identified using an anti-Momorcharin antiserum and peptides corresponding to different regions of the protein. It is worth noting that the peptide 71–136 corresponds to the catalytic region of this enzyme, indicating the possibility of a neutralization effect by any antibody binding within this area.

**Figure 3:**
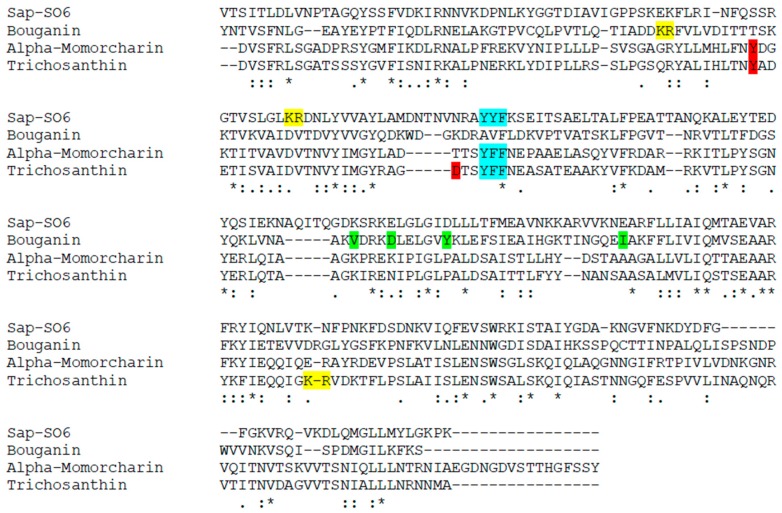
Multiple sequence alignment on RIPs for which there are experimental data on immunogenicity compared with Saporin. In green the four residues identified in Bouganin, in cyan the YFF (or YF) and in yellow and red the sequences identified in Thricosanthin. Alignment has been performed using CLUSTAL O(1.2.4) software (version 1.2.4., Conway Institute UCD Dublin, Ireland, 2017) [65].

Analysis of the primary structure (Figure 3) of Sap-SO6 compared to Bouganin, Tricosanthin and alpha-Momorcharin (the three Type I RIPs so far analyzed for immunogenicity) shows that a YYF or YFF sequence is present in all toxins except Bouganin, while the KR motif already identified in Trichosanthin is not significantly represented in conserved positions. Bouganin residues inducing immunological responses are not conserved among this group of Type I RIPs.

**Figure 4:**
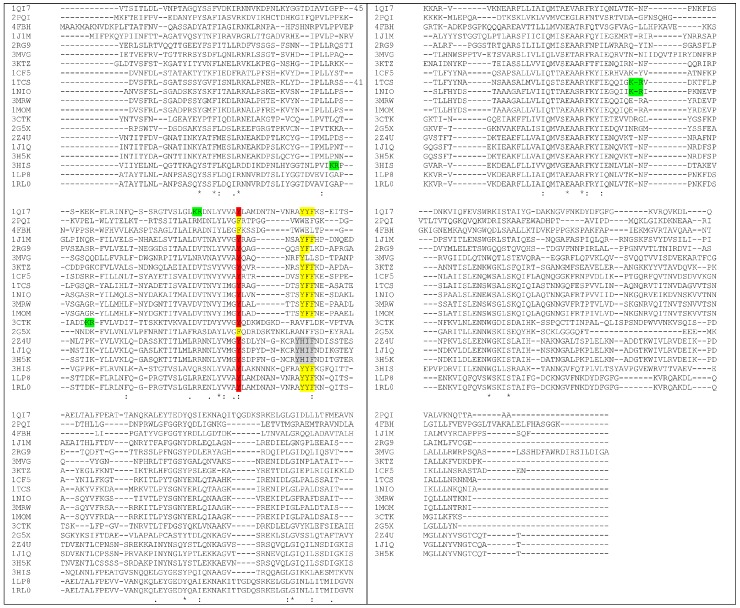
Sequence alignment of RIPs with known 3D structure (each one is indicated by the corresponding PDB code). YFF or similar motifs are shown in yellow. KR motif is shown in green. Tyrosine 76 (numbers refer to Saporin sequence, 1QI7) that can have a role in maintaining Y88 in position (see also below) is shown in red. Only three RIPs have F in this position. Alignment has been performed using CLUSTAL O(1.2.4) software [65]. PDB codes correspond to: 1QI7 Saporin SO6 from *Saponaria officinalis* seeds 2PQI RIP from maize; 4FBH RIP from barley seeds; 1J1M Ricin A-Chain; 3MVG RIP *Iris hollandica*; 3KTZ GAP31; 1CF5 β-Momorcharin from *Momordica charantia*; 1TCS Tricosanthin from *Trichosanthes kirilowii*; 1NIO β-Luffin from *Luffa cylindrica*; 1MOM Momordin from *Momordica charantia*; 3CTK Bouganin *Bougainvillea spectabilis*; 2G5X Lychnin from *Lychnis chalcedonica*; 2Z4U PD-L4 from *Phytolacca dioica*; 1J1Q Pokeweed Antiviral Protein from Seeds (PAP-S1) from *Phytolacca americana*; 3H5K PD-L1 from *Phytolacca dioica*; 3HIS Saporin-L1 from *Saponaria officinalis* leaves; 1LP8 Dianthin from *Dianthus caryophyllus*.

By means of structural alignment of the Type I RIPs (Figure 4) it can be seen that the YFF motif is largely conserved among these proteins even though those from *Phytolacca americana* and *Phytolacca dioica* (2Z4U; 1J1Q; 3H5K) share a peculiar YHIF motif in the same region.

**Figure 5:**
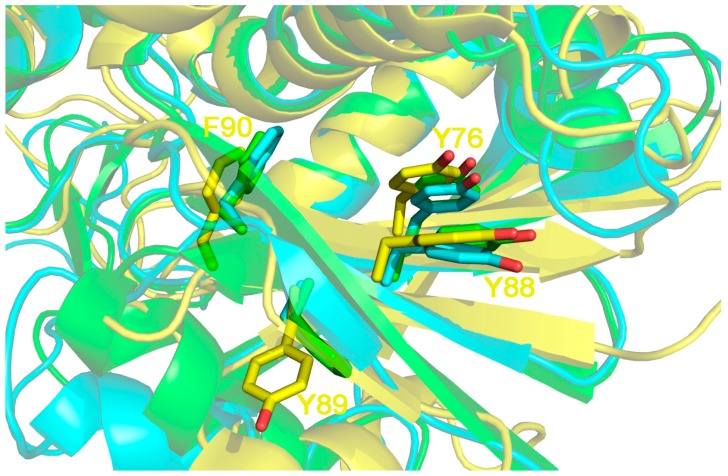
3D structure superposition between Sap-SO6 from *Saponaria officinalis* (pdb code: 1QI7; in yellow), Ricin chain A from (RTA) *Ricinus communis* (pdb code: 1J1M, in cyan) and Thricosantin from *Trichosantes kirilowii* (pdb code: 1TCS; in green) [9,13,15]. Residue numbering is according to Sap-SO6 structure. The Y[Y,F]F motif on the first tyrosine of the motif (Y88) is kept in place by a pi––pi interaction with Y76 on the β4 element. The presence of the latter is strictly correlated to that of the motif. Both Y76 and Y88 belong to 2 adjacent epitopes predicted, in the case of Sap-SO6, to be crucial for MHCII binding by the PropRed program [66] and are not directly involved in enzyme activity.

If the structural localization of the three residues is analyzed, (Figure 5) it can be seen that by comparing Sap-SO6, Ricin A chain and Thricosanthin, the first residue (Y88 in Sap-SO6) is always present and kept in position by another tyrosine (Y76 in Sap-SO6) conserved in almost RIPs (see Figure 5).

Thus, taking into account what is observed for Trichosanthin, it is reasonable to suppose that this conserved motif may be safely mutagenized in other plant RIPs including Saporin, resulting in a potential alteration of immunological epitope(s) without affecting the catalytic activity. This might therefore be considered as a good starting point for the de-immunization of plant RIP toxins in general.

**Figure 6:**
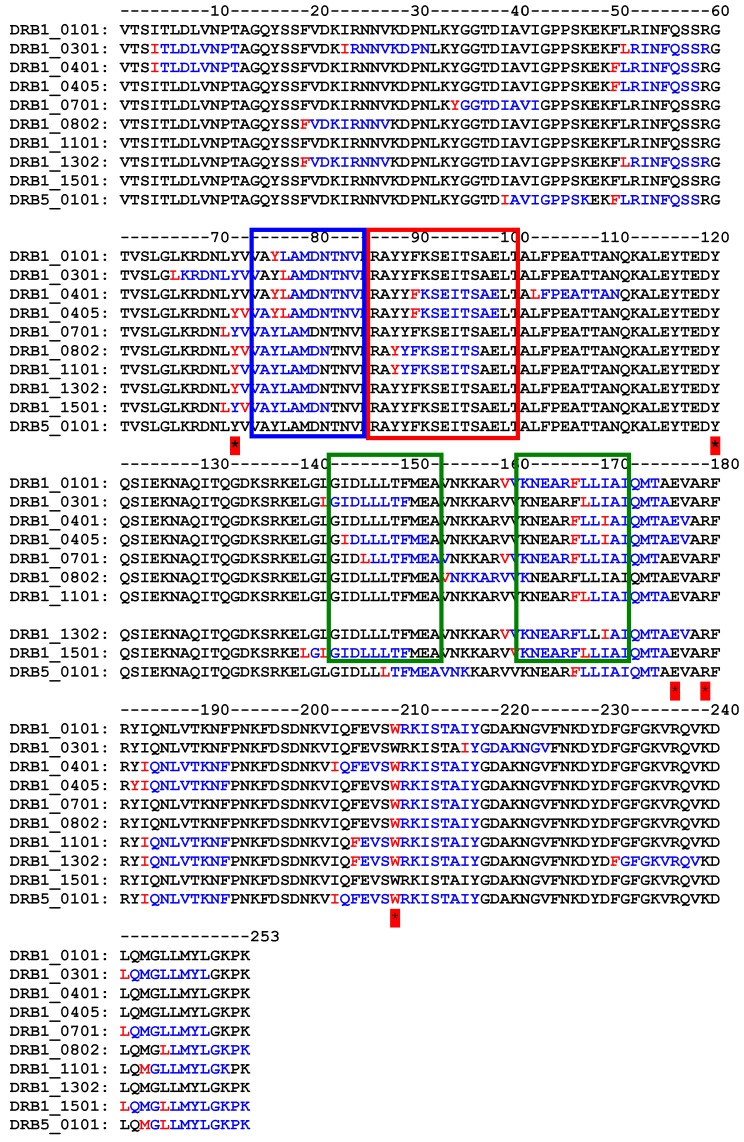
Immunogenic MHCII peptide prediction for Sap-SO6 (1QI7) on some of the more frequent alleles. Y72, Y120, E176, R179, W208, residues belonging to catalytic site are marked with * YYF sequences (box in red) as identified in Trichosanthin and regions corresponding to critical residues in Bouganin (box in green) are shown. PropRed program [66] shows immunogen peptides in blue and mark the first critical residue of immunogenic sequence in red.

The precise prediction of immunogenic epitopes eliciting immune response in humans is a complex issue, because of the presence of allelic variants of MHC class II. However, if the analysis is restricted to the most diffuse allelic forms of MHC class II (according to [67,68]) it can be observed that the computer analysis application PropRed [66] shows for Sap-SO6 (Figure 6), that there are a number of peptide sequences in the region 85–100 that are predicted to be recognized by the MHC class II alleles DRB_0401, DRB_0405, DRB_0802 and DRB_1101 containing the YYF sequence analyzed above. Particularly in the case of DRB_0802 and DRB_1101, Y88 is reported as the critical residue for the binding site recognition (see Figure 6, red box). The tyrosine residue (Y76 in Sap-SO6 and Y75 in Trichosanthin) adjacent to and making contact with Y88 in Saporin and Y81 in Tricosanthin, is in both cases included as an antigenic peptide predicted to be recognized by many varieties of allelic MHC class II molecules (see Figure 6, blue box). Furthermore, it can be seen that among the residues involved in the catalytic activity of Sap-SO6, only W208 has been included as potential MHC class II binding site. Since the catalytic site of Sap-SO6 and plant RIPs (see Figure 2) is deeply embedded in the protein matrix, it is unlikely that this peptide would be available for recognition by antibodies raised against this sequence in the immune response to the circulating toxin.

It should be further emphasized that, notwithstanding a difference in amino acid sequence between Sap-SO6 and Bouganin, the critical antigenic regions of Bouganin correspond (on the basis of the prediction made by the PropRed application) to peptides predicted to also be antigenic in Sap-SO6 (see Figure 6, boxes in green).

Thus, on the basis of the analysis of the experimental data available on Trichosanthin and Bouganin, and looking at both the structural position and the predictions made on MHC class II binding sites, we could hypothesize that in Saporin the antigenic critical regions are almost overlapping those of these two different Type I RIPs.

**Figure 7:**
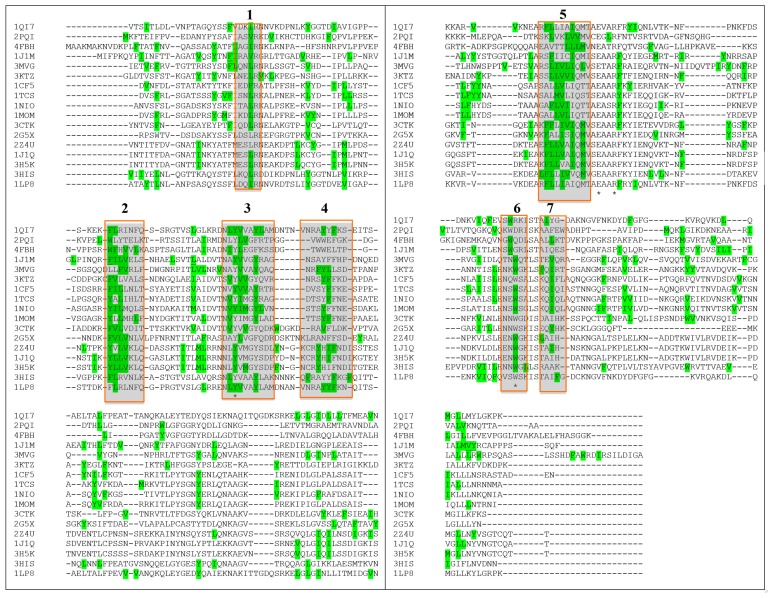
Multiple sequence alignment. In green the 1st aminoacid of MHCII peptides predicted by the program PropRed [66] within 3% threshold. * indicates the position of a catalytic residue. Alignment has been performed using CLUSTAL O(1.2.4) software [65]. PDB codes correspond to: 1QI7 Saporin SO6 from *Saponaria officinalis* seeds 2PQI RIP from maize; 4FBH RIP from barley seeds; 1J1M Ricin A-Chain; 3MVG RIP *Iris hollandica*; 3KTZ GAP31; 1CF5 β-Momorcharin from *Momordica charantia*; 1TCS Tricosanthin from *Trichosanthes kirilowii*; 1NIO β-luffin from *Luffa cylindrica*; 1MOM Momordin from *Momordica charantia*; 3CTK Bouganin *Bougainvillea spectabilis*; 2G5X Lychnin from *Lychnis chalcedonica*; 2Z4U PD-L4 from *Phytolacca dioica*; 1J1Q Pokeweed Antiviral Protein from Seeds (PAP-S1) from *Phytolacca americana*; 3H5K PD-L1 from *Phytolacca dioica*; 3HIS Saporin-L1 from *Saponaria officinalis* leaves; 1LP8 Dianthin from *Dianthus caryophyllus*.

Furthermore, if the prediction for antigenic sites in a larger number of plant RIPs is compared (between those whose 3D structure is known) some common features can additionally be observed. As shown in Figure 7, if the position of the first amino acid of each predicted immunogenic peptide is analyzed, a discrete number of clusters can be observed (numbered from 1 to 7).

**Figure 8:**
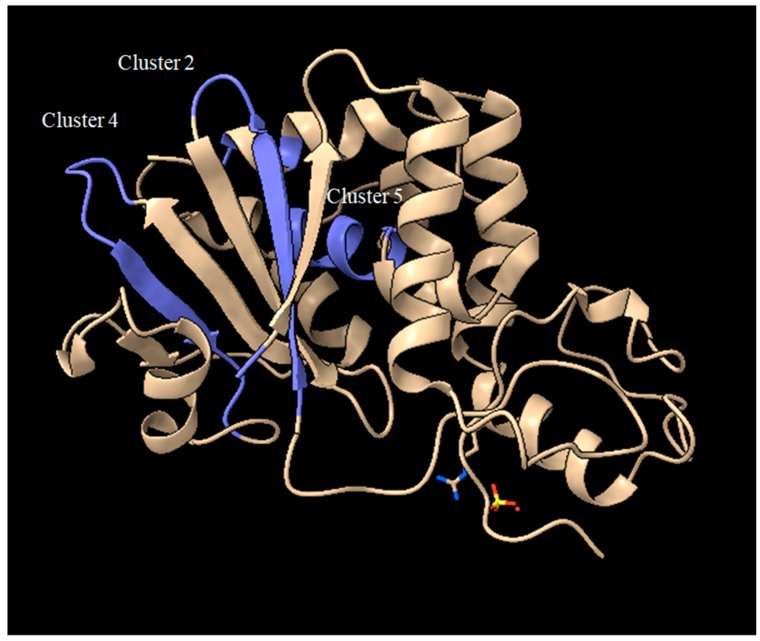
Crystal structure of Sap-SO6 (PDB: 1QI7) highlighting the position of clusters 2, 4, 5 from the analysis of Figure 7.

Among these, if we exclude those containing the key catalytic residues and those represented by a single amino acid, we can focus on clusters 2, 4 and 5. Looking at the position of peptides originating from the first amino acid in the cluster (typically 9 aminoacid peptides), for Sap-SO6 (see Figure 8) we can observe that both clusters 2 and 4 contain residues facing the loops that are exposed to the external part of the toxin, while cluster 5 is embedded in an alpha-helix that spans over an internal surface.

Thus, it could be inferred that residues present in the two loops exposed in clusters 2 and 4 should be investigated as potential candidates for de-immunization of this toxin.

## 5. Vascular Leak Syndrome: How Could We Modify Saporin Behavior?

Vascular leak syndrome (VLS) may occur due to the nonspecific binding of the plant toxic domains to vascular endothelial cells [69,70]. VLS is characterized by interstitial edema, hypoalbuminemia, weight gain, and in the most severe cases, hypotension and potentially fatal pulmonary edema. Vitetta and colleagues showed that ricin A (RTA) chain contains a consensus aminoacid sequence “X-Asp-Y”, that is also found in Interleukin-2, where “X” could be the non-polar aminoacids Leu, Ile, Gly or Val and “Y” could be Val, Leu or Ser constituting an oligopeptide sequence which may induce vascular damage to human endothelial cells in vitro by binding to integrin receptors.

Vitetta’s group first addressed this problem during the clinical treatment of cancer patients with RTA-based immunotoxins [71,72]. They used native deglycosylated ricin A chain and observed that dgRTA ITxs often caused a marked increase in vascular permeability leading to diffuse edema and organ failures [69,70,71,72]. This syndrome was linked to the use of both of plant and bacterial toxins, as well as the cytokine IL-2 [73,74,75]. These different proteins were all shown to interfere with fibronectin adhesion process that subsequently hampered cell-cell and cell-matrix interactions [69]. Vitetta and co-workers showed that peptides derived from RTA or from the bacterial toxin PE38, derived from *Pseudomonas aeruginosa*, when linked to a monoclonal antibody (anti-CD22 [70]), induced HUVEC cell mediated toxicity. Molecular modeling showed that the X-Asp-Y motif was mainly exposed on the surface of the RTA molecule [76]. A very similar motif is also shared by the viral disintegrins, which disrupt the function of integrin receptors [77]. Vitetta’s group produced a series of RTA mutants, and identified an Asn 97 to Ala mutation, in a region flanking the putative VLS-motif, that caused significantly less VLS in mice [71,76,78,79]. 

Peptides with altered or deleted “LDV” sequence in RTA were in contrast unable to cause damage to HUVEC cells. Further experimental work also identified other flanking structural amino acids as potentially contributing to the observed toxicity, while a mutagenesis strategy was been adopted to reduce the VLS properties of dgRTA by changing Arg48 and Asn97 that are in close proximity to the LDV sequence in the crystal 3D-structure of RTA [76]. Aspartic acid D75 (the central aminoacid of the LDV sequence) instead appeared to be a critical residue for RTA cytotoxicity and could not be mutated and, thus R48A, L74M, V76A, V76M and N97A mutants were considered as alternative options. The first results obtained introducing these single mutations showed that R48A and N97A mutants retained RTA full catalytic activity but did not elicit pulmonary leak syndrome in a mouse model. The various mutated RTAs were used to construct anti-CD22 immunotoxins that were then tested in a mouse lymphoma model [76]. The immunotoxin containing R48A showed very similar pharmaco-kinetic properties to those of the dgRTA-based immunotoxin while its therapeutic index was even better than the original dgRTA-antiCD22. Thus, it was conclusively demonstrated that by acting on LDV and its surrounding aminoacid residues it was possible to reduce VLS and thus, improve the therapeutic index increasing the potential clinical application of RTA-based ITxs. 

**Figure 9:**
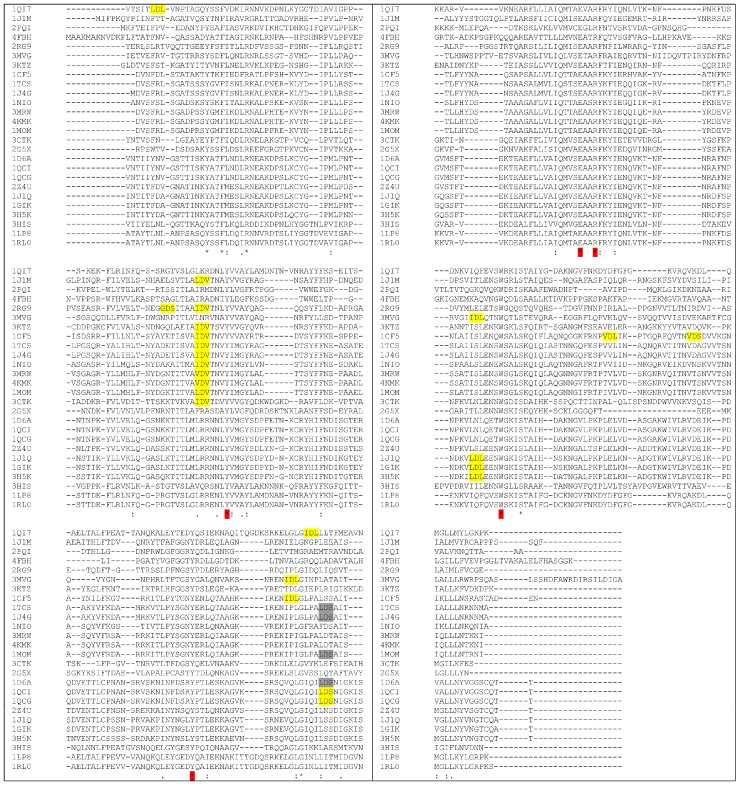
Multiple sequence alignment of Type I RIPs. “X-Asp-Y” motif is shown as highlighted in yellow. Alignment was performed using CLUSTAL O(1.2.4) software [65]. PDB codes correspond to: 1QI7 Saporin SO6 from *Saponaria officinalis* seeds; 1J1M Ricin A-Chain; 2PQI RIP from maize; 4FBH RIP from barley seeds; 2RG9 *Viscum album* mistletoe lectin I; 3MVG RIP *Iris hollandica*; 3KTZ GAP31; 1CF5 β-Momorcharin from *Momordica charantia*; 1TCS Tricosanthin from *Trichosanthes kirilowii*; 1J4G Tricosanthin from *Trichosanthes kirilowii*; 1NIO β-luffin from *Luffa cylindrica*; 3MRW Ribosome Inactivating protein from *Momordica balsamina*; 4KMK Ribosome Inactivating protein from *Momordica balsamina*; 1MOM Momordin from *Momordica charantia*; 3CTK Bouganin *Bougainvillea spectabilis*; 2G5X Lychnin from *Lychnis chalcedonica*; 1D6A *Pokeweed antiviral protein* (*PAP*) from *Phytolacca americana*; 1QCI Pokeweed antiviral protein (PAP) from *Phytolacca americana*; 1QCG Pokeweed antiviral protein (PAP) from *Phytolacca americana*; 2Z4U PD-L4 from *Phytolacca dioica*; 1J1Q Pokeweed Antiviral Protein from Seeds (PAP-S1) from *Phytolacca americana*; 1GIK Pokeweed antiviral protein (PAP) from seeds of *Phytolacca american**a***; 3H5K PD-L1 from *Phytolacca dioica*; 3HIS Saporin-L1 from *Saponaria officinalis* leaves; 1LP8 Dianthin from *Dianthus caryophyllus*; RIP Dianthin 30 from *Dianthus caryophyllus*.

**Figure 10:**
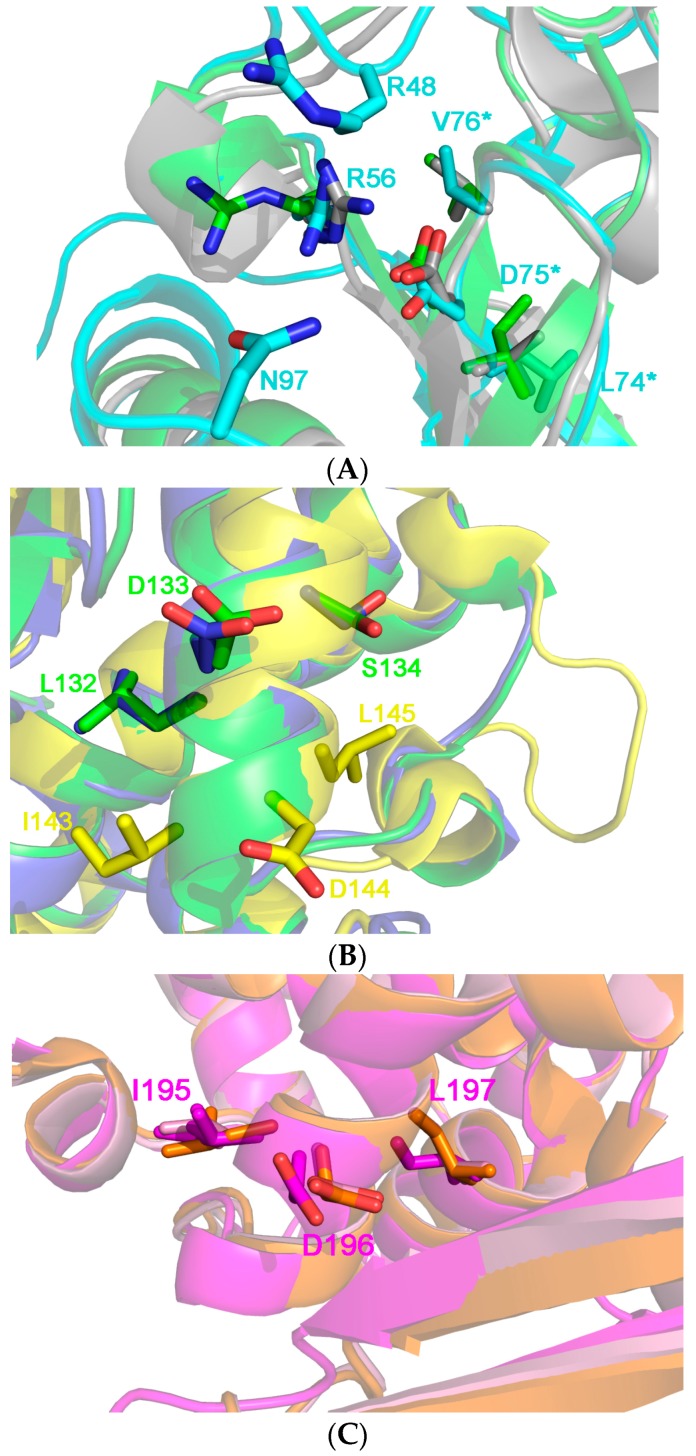
(**A**) 3D structure superposition between Ricin chain A from *Ricinus communis* (pdb code: J1M, in cyan), Thricosantin from *Trichosantes kirilowii* (pdb code: 1TCS; in green) and ribosome inactivating protein from *Momordica balsamina* (pdb code 4KMK: in gray) [13,15]. The structures have been chosen as representative of Cluster 1 (11 RIPs), where the RIPs are characterized by the conserved [L, I, V] D, V motif at the end of the β5 element. Residue numbering is in accordance to the Ricin A structure. L74, D75 and V76, the residues belonging to the motif are highlighted by an asterisk at the right of the figure. The two residues, i.e., R48 and N97, whose mutants do not elicit the vascular leak syndrome in animal models are not conserved both in the sequence and in structure in the other proteins of the cluster. R56 directly interacts with D75 probably keeping it in place and its position is conserved in most of the protein belonging to Cluster 1. We speculate that this residue is crucial to maintain the architecture of this protein portion, which is adjacent to the active site pocket of the toxin; (**B**) 3D structure superposition between Thricosanthin from *Trichosantes kirilowii* (pdb code: 1TCS; in green), Ribosome inactivating protein from *Momordica charantia* (pdb code 1MOM: in blue) and Sap-SO6 from *Saponaria officinalis* (pdb code: 1QI7; in yellow) [9,14,15]. The former structures have been chosen as representative of cluster 2 (6 RIPs), whose members are characterized by the L,D,S motif in the αhelix 4. Residue numbering is according to the structure of Thricosanthin. The IDL motif present in the Saporin is peculiar, and it is found one helix-turn before to those of Thricosantin and the RIP from *M. charantia*; (**C**) 3D structure superposition between RIP from *Phytolacca americana* chain A (pdb code: 1J1Q; in orange), RIP from *Iris hollandica* chain A (pdb code 3MVG: in purple) and RIP from *Phytolacca dioica* chain A (pdb code: 3H5K; in pink) [80]. The structures belong to Cluster 3 (3 RIPs), whose members are characterized by the I, D, L motif in the αhelix 6. Residue numbering is according to the structure of RIP from *Phytolacca americana*.

Figure 9 shows the comparison of several aminoacidic RIP sequences demonstrating that many Type I RIPs share the same (x)D(y) motif, with a large group of them strikingly maintaining this VLS motif in a similar position as found in RTA. Other plant RIPs, in contrast, show a somehow different localization of this motif along the length of the polypeptide and some other plant toxic domain also show a potential double VLS-eliciting peptide. In particular, seed-extracted Sap-SO6 has an “LDL” sequence at the N-terminal and an “IDL” sequence closer to its C-terminal region. On the basis of our sequence alignments, we can speculatively cluster the analyzed RIPs sequences into three different groups, the first one (including Ricin A chain (RTA), Trichosanthin (TC) and the RIP from *Momordica balsamina*, see Figure 10A sharing LDV (or similar) sequences with Ricin A chain. The two residues that have been mutated in RTA to eliminate VLS (i.e., R48 and N97) are not however, found conserved among RIPs of this first group (see Figure 9). Therefore it is difficult to predict whether mutants at the corresponding positions in other plant RIPs of the same group could be effective in reducing potential VLS-derived problems.

What can instead be clearly noted in Figure 10A, is that residue R56 is well conserved and is positioned to maintain D75 in RTA structure; this suggestion that this particular position, closely adjacent to R48 and N97, could be explored as a potential residue in this cluster of RIPs to be mutated as a strategy to reduce VLS.

If the crystal 3D-structures of other plant RIPs belonging to cluster 2 (including Trichosanthin and Momordin) and Sap-SO6 (see Figure 10B) is examined we may see that the LDS motif is located on alpha helix 4 which is well exposed to the external surface and thus, potentially capable of binding to integrins that may consequently result in VLS. Sap-SO6 has an unusual “IDL” motif located just one helix-turn before the L,D,S sequence. Sap-SO6 possesses another L,D,L motif located at the N-terminus of the mature protein, and we can therefore speculate that Saporin possesses two other potential binding sites for integrins. No further conserved residue within this region is detectable, thus any prediction of potentially mutagenic sites available to reduce Sap-SO6-induced VLS remains speculative. The only conclusive observation we can assume is that since location of the VLS sequence in this second cluster is distant from the key catalytic residues within the catalytic cleft, their mutagenesis should not affect the N-glycosidase activity of the molecule. 

A third cluster of RIPs (including PAP, PD-L1 and the RIP from *Iris hollandica*, (see Figure 10C) has an L,D,L motif in the α-helix 6. In the case of the latter RIPs it is not possible to identify any other conserved residue around the motif on which acting by selective mutagenesis to reduce VLS. In this case, a first approach could be directed to mutagenize the L,D,L motif itself.

Sap-SO6 displays unusual structural motifs when compared to other plant RIPs and this therefore demands a specific mutagenesis strategy, in order to validate the actual contribution of these two sequences in the genesis of VLS.

## 6. Understanding the Intracellular Fate to Improve the Efficacy of Saporin-Based Chimeras

An important area of investigation is the search to improve the therapeutic index of Saporin-based targeted toxins by increasing their tumor target specific cytotoxicity. Several different co-treatments are described in the literature aiming to maximize the toxins cytotoxic therapeutic effect without increasing off-target toxicity. Understanding the intracellular fates of the Saporin molecule and the effects of the various cytotoxicity enhancers on these steps will be essential to the clinical development of these different approaches. 

**Figure 11:**
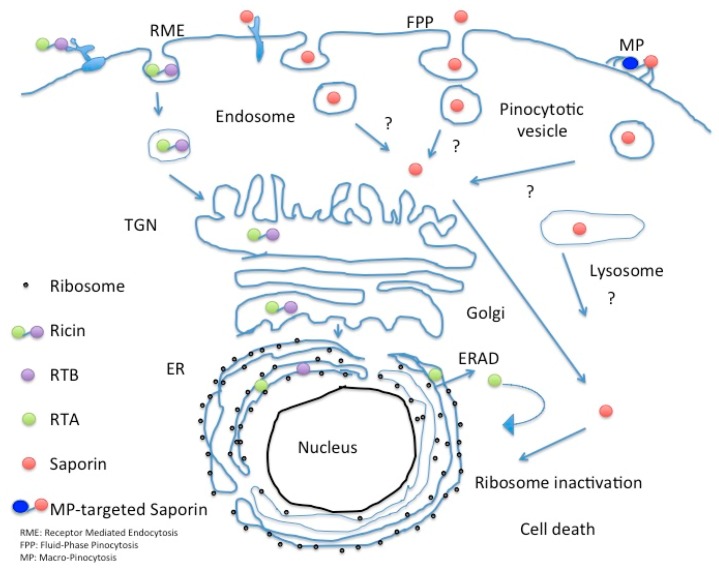
Intracellular fate of Saporin in comparison to Ricin holotoxin.

The route followed by Ricin holotoxin in intoxicated cells has been intensively studied, as well as that followed by the bacterial RIP Shiga toxin. Sandvig and colleagues pioneering work first showed in 1992 that the latter exogenous bacterial toxin reached Trans Golgi network (TGN) and subsequently also the ER when A431cells were treated with butyric acid by following a retrograde transport route [81] to be finally retrotranslocated to the cytosol, in order to access target ribosomes and inhibit protein synthesis. Retrograde trafficking routes from early, recycling and late endosomes seem to exist in parallel [82]. This landmark paper established the existence of a transport connection between plasma membrane/endosomes and TGN/Golgi complex/endoplasmic reticulum. Ricin follows a similar Golgi retrograde transport pathway, and it has been demonstrated that this toxin exploits ER chaperones (calnexin/calreticulin) cycling between this compartment and the TGN to reach the ER where A and B chain of the holotoxin dissociate due to the action of Protein Disulfide Isomerase (PDI) [83] and thioredoxin-reductases that reduce the disulfide bond between the A and B chains [5]. Free RTA then mimics a misfolded protein and is therefore targeted for proteasomal degradation [84,85,86] by interacting with EDEM (ER degradation-enhancing α-mannosidase I-like protein 1) and EDEM-2 both of which are key players in redirecting aberrant proteins for ERAD (ER-associated protein degradation). Once in the cytosol the A chain undergoes a rapid refolding and depurinates ribosomal 28S RNA [86]. Type I RIPs are believed to pass through the endo-lysosomal compartment but as shown for Sap-SO6 they likely bypass the Golgi-mediated transport route (see Figure 11) [84,85,86].

Cellular toxicity of RIPs may be mainly attributed to their rRNA depurination activity, which in turn induces a “ribotoxic stress response” characterized by activation of several protein kinases. Generation of reactive oxygen species (ROS) in response may also cause oxidative stress to cells, which might be one of the key factors in inducing apoptosis through the mitochondrial pathway [87]. It has been demonstrated that Saporin induces cell death via the mitochondrial or intrinsic apoptotic pathway [88]. The capability of RIPs to induce cell death by apoptosis has also been demonstrated using various in vitro and in vivo models [89]. 

Saporin cytotoxicity has been shown to act in a caspase-dependent manner since inhibition of caspase-3 also results in the inhibition of apoptosis. Furthermore, it has been shown that Saporin-SO6, unlike many other RIPs, did not necessarily depend only on N-glycosidase activity for cellular toxicity, it having been reported that internucleosomal fragmentation of DNA may also contribute to cellular damage [90]. However this putative DNase-like activity was not confirmed using a recombinant Saporin seed isoform [91]. Finally, the apoptosis onset for Saporin has also been suggested to occur before any significant inhibition of protein synthesis [92].

Escape of toxin across the endo-lysosomal membrane system represents a major bottleneck that prevents the efficient translocation of Type I RIPs from the lumen of intracellular vesicles to the cytosol. Several endosomal escape enhancers have been investigated with the goal of disrupting the intracellular vesicular membrane without increasing any non-specific off-target toxicity. Other such examples include the use of lypopolyamines or DMSO, both of which lead quantitatively to an increased access of Saporin to the cytosolic compartment with a commensurate increase in cytotoxicity. However, neither of these compounds enhances the cytotoxic activity of RTA, probably reflecting the different retrograde trafficking pathway this toxin takes through the TGN and ER [93,94].

A very promising means to augment Saporin-based targeted toxins is the use of glycosylated triterpenoid saponins (derived from a wide variety of plant sources including *Saponaria officinalis*). Weng et al. [95,96] reported that SA1641, a saponin derived from Gypsophila species (Baby’s Breath) may stimulate the endocytic uptake of Saporin by target tumor cells. 

Another saponin species, SO-1861 from the roots of *Saponaria officinalis* also significantly augmented the therapeutic efficacy of EGF-Sap (Sap3-EGF, i.e., Saporin isoform 3 fused to epidermal growth factor) against the EGFR-expressing TSA mammary carcinoma cell line TSA growing in syngeneic mice [97]. In this study, the use of 0.1 μM SO-1861 alongside EGF-Sap reduced the dose of targeted toxin Sap3-EGF required to achieve a therapeutic response against EGFR expressing TSA [98].

Saponin SA1641 (extracted from *Gypsophila paniculata* L.) was used as a co-treatment together with a chimeric Saporin protein, containing the PDZ domain from hCASK (Human calcium/calmodulin-dependent serine protein kinase) binding extracellular CD98 as the targeting moiety. This antigen is used as a marker for several human tumors and is particularly considered as a negative prognostic marker in human glioblastoma. The recombinant fusion toxins with either one (h(CASK)-Saporin) or two (h(CASK)_2_-Saporin) PDZ domains were tested against several glioblastoma cell lines. Saponin SA1641 was able to increase the toxicity of Saporin and h(CASK)_2_-Saporin chimera in U87 Glioblastoma cell line (IC_50_ < 10^−11^ M and << 10^−12^ M respectively), and allowed hCASK-Saporin to reach an IC_50_ of 10^−10^ M [99,100]. 

Lombardi and co-workers compared the performances of anti CD22 single chain variable fusion constructs/scFv to identify novel recombinant single chain antibodies showing nanomolar affinity for the CD22 antigen able to deliver, either Saporin or *Pseudomonas aeruginosa* PE40 bacterial-derived toxin, to tumor cells and found comparable cytotoxicities between the plant or bacterial derived ITxs (see the section below) [91,101]. *Saponinum album* saponins (SA) augmented the PE40-based ITx by 170-fold while the Saporin-based ITx was augmented by more than 6 orders of magnitude following this co-treatment. This great difference in the saponin enhancer effects on PE40-ITx versus Saporin-based ITxs reflects the preferential interaction of some Type I RIPs with these triterpenoid glycosylated saponins (D.J. Flavell, unpublished results). Holmes et al. [102] investigated the augmentative effects of *Saponinum album* saponins on Saporin-based ITxs constructed with antibodies directed against CD7, CD19, CD22, CD38 and CD71 on three different human leukemia or lymphoma cell lines, but found considerable cell line and target molecule variation on the extent of augmentation observed, ranging from just 174-fold to greater that 31.5 million-fold for Daudi lymphoma cells targeted via CD19 antigen. These authors also showed that a proportion of the augmentative effect of SA on ITx cytotoxicity was off-target, a problem that was partially resolved by scheduling exposure to ITx first followed by SA second. Smith et al. [103] have further shown that augmentation of Saporin-based immunotoxins by SA is cholesterol-dependent and furthermore can be abrogated by pharmacological inhibitors of endocytosis, endo-lysosomal acidification and actin polymerization. These various observations led Holmes et al. [102] to hypothesize that saponins form complexes with the RIP Saporin within the acidic endo-lysosomal compartment and that at low pH the complex may assume membranolytic activity thus damaging the endo-lysosomal limiting membrane allowing toxin escape into the cytosol. 

As a general rule endosomal escape enhancers: (i) should not exert any appreciable toxicity *per se* for the target cells; (ii) their co-adjuvant role requires their concomitant presence at the site of dislocation during the passage of the toxin “en route”—to its final destination (iii) these agents should not facilitate toxin cytosolic entry into off-target cells.

These optimal conditions should be ideally tested in in vivo systems. Unfortunately, most of the data published using endosomal escape enhancers to date have been examined mostly in vitro. Further studies in in vivo model systems are therefore urgently required to validate the in vitro results necessary for pre-clinical development. Some authors have however, demonstrated efficacy of endosomal escape enhancers in animal models [104]. For a comprehensive review of endosomal escape enhancers used to potentiate plant RIP-based ITxs please refer to Fuchs et al. [105].

Another innovative methods to enhance the delivery and uptake of therapeutic agents, as Saporin, is the encapsulation of drugs using specific exosomal membranes modified with different peptides including a cationic pH-sensitive fusogenic peptide known as the GALA peptide [106]. These viral-derived peptides are capable of disrupting of the endosomal limiting membrane, leading to a more efficient cytosolic release of protein toxin cargo. Nakase and coworkers used GALA peptides, one with the sequence WEAALAEALAEALAEHLAEALAEALEALAA (amino acid one letter names) designed to mimic a viral specific fusion peptide sequence that mediates the escape of viral genes from the late endosome acidic lumen into the cytosol. The molecular structure of the GALA peptide is converted from a random coil to a helix when the pH drops from 7.5 to 5.0, as it passes from early to late acidified endocytic vesicles. This ultimately leads to membrane fusion and release of luminal cargo to the cytosol. This technique was successfully used to deliver Saporin to the cytosol of HeLa cells, leading to an efficient induction of cytotoxicity in HeLa targeted cells [106].

## 7. Macropinocytotic Pathways as Possible Efficient Entry Paths?

Macropinocytosis is up regulated in several cancer cells and is a more efficient internalization pathway capable of bulk uptake of fluids from the external milieu. Macropinosome vesicles are on average of 200–300 nm diameter and additionally provide a further opportunity for nano-delivery approaches. Using an automated microscopy-based imaging platform to identify phage antibodies co-localizing with macropinocytotic markers, Ruan and co-authors screened their single chain antibody libraries enriched in internalizing antibodies by a laser capture micro-dissection selection, which bound preferentially to tumor cells [107]. These workers have selected phage displayed antibodies that favor this unusual entry route which were re-engineered into full length humanized IgG molecules and showed specific targeting to the ephrin type A-receptor 2, a molecule identified as being involved in tumor invasion and metastases making this an interesting potential candidate for targeted cancer therapies [108]. Cancer cell lines overexpressing ephrin type A-receptor 2 were used to show efficient killing by a derivatized biotinylated IgG antibody (termed HCA-F1) directed against the ephrin type A receptor followed by reaction with a streptavidin-conjugated Saporin to produce a specific ITx that killed DU145 prostate cancer cells with an Ec50 of around 19 pM after 96 h exposure. The same ITx was found to be ineffective against receptor negative cells. The authors observed that either fusions made with sc-Fv or with the intact IgG can be used for secondary screenings, since no major differences were observed in the internalization patterns between these two antibody formats. In addition, since the epitope bound by this novel HCA-F1 anti-EphA2 antibody shows cross-species specificity, the toxicity profiles could therefore initially be evaluated in small rodents. This is the first example of a phage antibody library that allows for the screening of macropinocytotic antibodies and could prove a useful tool to select new agents targeting tumor cells that exploit the macropinocytotic entry route (see Figure 11). 

## 8. Co-Treatments Can Improve Saporin-Based ITx

Antineoplastic treatments with ITx are often only partially effective or even completely ineffective when only one ITx targeting a single molecule on the tumor cell is administered. To enhance and optimize the anti-cancer potency, multiple ITx could be administered simultaneously. As a “proof of principle” of the possibility of targeting two overlapping but distinct subpopulations of cells by using a secondary immunotoxin treatment is the dual treatment used against Glioblastoma Multiforme (GMB) by combining the use of the “Mab-Zap” Saporin immunotoxin system developed by Kohl and co-workers [109]. The “Mab-Zap” System is a system that identifies potential targeting antibodies (mouse monoclonal) and a second anti-mouse antibody conjugated with a toxin (i.e., anti-IgG antibody that is toxin-coupled). An example of the application of this innovative system consists of monoclonal antibodies versus NG2 (Neuron-glia 2 (NG2), is a transmembrane chondroitin sulfate proteoglycan) and GD3A (GD3A, is a ganglioside expressed on cell surface of developing migratory glia) used together with sequential use of an anti mouse antibody-Saporin. Targeting these antigens found re-expressed in GBM produced ablation of both NG2 and GD3A-expressing cells, resulting in significant reduction in GBM cell viability as compared to the single epitope targeting as controls [110].

An interesting example of a co-treatment with a Saporin-based immunotoxin and anti-CD20 antibody was described by Flavell and colleagues [45]. These workers showed that by using the chimeric anti-CD20 antibody rituximab (Rituxan) in combination with the anti-CD19 immunotoxin BU12-Saporin targeted against Ramos cells (a human B-cell lymphoma cell line) a significantly greater therapeutic effect was obtained both in vitro (measured by cytotoxicity and apoptosis induction) and in vivo in a SCID mouse model of disseminated lymphoma (measured as disease-free survival). These authors concluded that a combined treatment with two immunotherapeutic reagents directed against two different B-lineage molecules on the target cell surface each with a different mode of cell killing resulted in an increase in both the potency and fidelity of the immunospecific attack on the lymphoma [45].

Rituximab has also been conjugated to seed extracted Saporin in combination with another anti-CD22 ITx containing Saporin. These two ITxs were created by chemical conjugation of the appropriate antibody to Saporin. Rituximab/Saporin and OM124/Saporin are respectively directed against CD20 and CD22, both antigens highly expressed on Non-Hodgkin’s lymphomas (NHLs) and found particularly expressed at high levels on normal mature B-cells and on a large population of B-lymphoma cells but absent in the normal tissues and hematopoietic stem cells. A way to increase the therapeutic efficacy of these two Saporin-based ITxs was obtained by the simultaneous administration of chemotherapeutic agents or cell inhibitors, such as the proteasome inhibitors PS-341, MG-132, or the purine analogue fludarabine [111].

Finally, a protocol termed 3BIT consisting of a combination of three different saporin-based ITx directed against CD19, CD22 and CD38 was reported by Flavell’s group [44]. The combination of all three immunotoxins was curative in one hundred percent of SCID mice bearing disseminated human Ramos lymphoma whereas treatment with individual ITxs or combinations of two ITxs only cured a proportion of the mice. The combination treatment with 3BIT is speculated to work because of heterogeneity of single antigen expression on Ramos lymphoma cells. By targeting three receptor molecules on the lymphoma cell surface simultaneously reduces the possibility to escape due to heterogeneity of expression of any individual target molecule. 

An interesting issue is correlated to the chemically linked immunotoxins, where both the number of toxins/antibody or the total number of both the components may affect the activity of the conjugate. A Saporin-Rituximab immunotoxin was obtained by chemically conjugating via a disulfide bond rituximab to Saporin to yield two immunoconjugate species, a HMW-ITx (Dimeric) and LMW-ITx (Monomeric) each of which had similar activity in inhibiting protein synthesis in a cell-free system. However, in two CD20^+^ lymphoma cell lines, Raji and D430B, the HMW-ITx was more cytotoxic than the LMW-ITx. Such a double ITx system (HMW and LMW-ITx) is suggested by the authors to be flexible in a potential clinical use, devoting the dimeric HMW use as endocytosis enhancer (for antigens with low internalization rate) or as an improved penetration system for solid tumors due to the reduced molecular size of the LMW ITx [112]. These authors did not however investigate the two ITxs in an experimental system in vivo to establish their toxicity, pharmacokinetics and therapeutic activity so it is difficult to know whether this approach will be likely to have any practical clinical benefit. 

Flavell and collaborators [36] showed that an anti-CD7-Saporin immunotoxin constructed with two Saporin moieties per antibody (2-mer) was more potent in vitro against the T-cell acute lymphoblastic (T-ALL) cell line HSB-2 that one constructed with only one Saporin moiety (1-mer). However, the 2-mer ITx proved significantly more toxic in vivo in a SCID mouse model of disseminated T-ALL and offered no therapeutic advantage over the 1-mer ITx. 

## 9. Enhanced Delivery through PCI-Phototherapy Approaches

The classical approach to site-specific drug delivery of a target-specific immunotoxin against tumors cells requires receptor-mediated endocytic uptake of the drug and efficient release of the therapeutic molecule from intracellular endocytic compartments, into the cytosol. This process has several limitations among which the low rate of penetration through the membranes of endocytic vesicles and/or compartments and the degradation of therapeutic macromolecules trafficked to lysosomes by proteolytic enzymes [113].

To at least partially circumvent this problem a light-based, an innovative technology has been developed termed photochemical internalization (PCI).

PCI may help to release mostly endocytosed macromolecules into the cytosol being based on the use of specific photosensitizers located within endocytic vesicles that, upon activation by light, induce the drug release due to photochemical-mediated rupture of endocytic membranes [113].

Weyergang and colleagues demonstrated that PCI significantly increases the toxicity of EGF–Saporin on the EGFR^+^ rat epithelial ovarian cancer cell line NuTu-19 cells approximately 1000-fold relative to EGF-Saporin treatment of cells without PCI [114].

The evolution of PCI technology as an in vivo treatment has been developed through the use of laser-controlled endosomal escape in localized tumors. An example of such an application is the treatment with a CD133-targeting immunotoxin AC133–Saporin (PCIAC133–Saporin) that co-localizes with the PCI-photosensitizer TPCS2a in CD133 positive cancer stem cells. After light exposure, cytosolic release of AC133–Saporin was induced in target cells with a concomitant increase in cytotoxicity. The advantages of this novel technology is the ability of PCI-photosensitizer to preferentially accumulate within tumor tissues, allowing the possibility to trigger the PCI photosensitizer in vivo locally in tumor tissue combined with the tumor-selectivity of the immunotoxin [115].

PCI is also valuable for treatment of CSCs expressing CD44, that characterize tumor cells highly resistant to ROS attack, rendering originally resistant cells to be more sensitive to chemo- and radiotherapy. CD44 has also been reported to be involved in multidrug resistance (MDR). Bostad and coworkers made an ITx using a biotinylated-CD44 mAb and an avidin-Saporin. After administration of the PCI-photosensitizer TPCS2a and ITx in seven different carcinoma and sarcoma cell lines an efficient and specific cytotoxicity in CD44-expressing but not in CD44-negative cancer cells was observed [116].

Another antibody whose cytotoxic performance is greatly improved by PCI is the commercially humanized HER2 mAb, trastuzumab. This FDA-approved antibody was used in chemotherapeutic treatment of HER-2 positive breast cancer. Unfortunately, the acquired resistance to trastuzumab treatment is a common outcome difficult to solve. A novel strategy to overcome this problem is by the use of PCI. Using biotinylated-Trastuzumab and avidin-Saporin a novel ITx was generated for PCI, and co-administered with PCI-photosensitizer TPCS2a to trastuzumab-resistant HER2^+^ Zr-75-1 cells prior to light exposure (i.e., “light after” procedure) that increase the cytotoxic effect because “light first” procedure also reduces the trastuzumab-induced HER2 endocytosis of ITx [117].

Vikdal and colleagues demonstrated that PCI can also increase the accumulation of free Saporin and subsequent enhanced PDT induced cytotoxicity in the HUVEC cell line with respect to HT1080 cell line (fibrosarcoma). This strategy can be used as an antivascular treatment in the anticancer therapy [118].

An alternative approach to overcome the potential barriers that can reduce the cellular uptake and intracellular release of Saporin is the use of generation four polyamidoamine (PAMAM) dendrimers carrier molecules. These molecular constructs are specifically capable of increasing the endocytic uptake, the passive tumor targeting, and could be utilized in the context of photochemical internalization (PCI) technology, facilitating the Saporin cellular uptake and cytosolic release [119].

Recently a PCI-based drug delivery system was applied to EpCAM positive cancer cells. A novel ITx was generated using an EpCAM-targeting mAb 3–17I chemically coupled to Saporin and when ITx was tested in vitro showed a potent and selective reduction of cellular viability, proliferative capacity, and colony forming ability in breast carcinoma, pancreatic adenocarcinoma, and colon adenocarcinoma cell lines [120].

## 10. Toxin Plasmid DNA Co-Transfections

As further innovative procedure under investigation consist of plasmid co-transfection using two toxins DNA sequences in an in vitro model. This technique, termed originally “Toxic gene therapy” (suicide gene therapy) has been successfully tested on various cancer cell lines (HeLa, U87, 9L, and MDA-MB-435) by using two gene toxins coding for the mature polypeptide inserted into two distinct mammalian gWIZ plasmids: pGEL (gWIZgelonin) and pSAP (gWIZ-Saporin). The co-transfection treatment in mammal tumor cell lines was achieved by using cationic polyethyleneimine (PEI) and exerted a potent and specific cytotoxicity [121].

## 11. Improvement of Saporin Chimera’s Production by Optimization of Recombinant Expression

Another further relevant aspect for the development of Saporin chimeras (and other plant RIPs chimeric toxins) as pharmaceutical agents is their efficient expression and proper folding of the nascent fusion polypeptide chain in a host cell. To express plant protein toxins and especially recombinant fusion chimaeras, based on Saporin or Type I RIPs it would be preferable to use a eukaryotic expression system, firstly because Type I RIPs such as Saporin, are secretory proteins and secondly because correct folding by the cellular quality control system is most readily achievable in the microenvironment of the endoplasmic reticulum (ER). However, exogenous protein toxin expression may trigger autointoxication issues. The principal way in which *Ricinus communis* protects itself from self-intoxication is to produce a long inactive precursor where the nascent polypeptide is efficiently co-translationally inserted inside the lumen of ER to avoid ricin mislocalization in the cytosol and inactivation of target ribosomes [122]. When orphan ricin A polypeptide chains are expressed in Tobacco leaf protoplasts they are retained in the ER and then retrotranslocated to the cytosol for proteasomal degradation [123]. The Saporin plant precursor instead follows a quite different fate in this plant model system. In contrast to what was observed for Ricin A chain, Saporin polypeptides were found to be efficiently secreted into the incubation media while the observed protoplast’s intoxication was demonstrated to be due to an ER stress-dependent pathway. Saporin cytotoxicity apparently involved a few toxin molecules that were at least first partially inserted into ER membranes [124], thanks to the N-terminal signal peptide that may also act as an ER-stress responsive element [125]. Other Type I RIP signal peptide(s) may also act similarly as ER stress-sensors [87]. It is, therefore, important to avoid the use of endogenous N-terminal sequences in fusion constructs to be expressed in heterologous eukaryotic systems. To date bacterial, model plant cells and more recently, yeast strains have been used to produce RIPs or RIP-based chimeric fusion proteins. However, common problems faced during recombinant production of Type I RIPs or their derived chimaeras resides in their intrinsic toxicity towards host ribosomes. Initial attempts to express recombinant Type I RIPs in *E. coli* were also found problematic, because, upon induction of RIP expression the bacterial growth rate was significantly impaired, as reported earlier in the case of Mirabilis antiviral protein [125], PAP [126], dianthin [127] well as Saporin [128]. More recently a Saporin L1/L3 vacuolar leaf variant could not even be expressed in a bacterial system due to its high toxicity [129].

Although toxin expression could be more tightly regulated by employing the *E. coli* strain BL21 (λDE3) pLysS to give satisfactory yields, issues such as endotoxin A contamination and processing of the insoluble bacterial pellets remained problematic [18]. Strikingly, while Saporin L3 was found too toxic even during the simple propagation procedures of plasmids containing the RIP’s DNA in host bacteria, L3 variant was reported, instead to be expressed only in microbial *P. pastoris* under an alcohol-oxygenase (AOX1) tight regulated promoter by inserting the prepro-alpha factor signal peptide at the NH2-terminus of Saporin L3, as we also previously reported to obtain high level of expression of Saporin seed isoform in *P. pastoris* [91]. The choice of a tight inducible regulated system such as AOX-1 together with an efficient signal peptide such as the one of the preproalpha-factor or alternatively by using those naturally present in endogenous ER chaperones such as Immunoglobulin binding protein (BIP) or protein disulfide isomerase (PDI) is also of utmost importance to avoid any toxin mislocalization into the eukaryotic cytosol. This microbial protein expression system has shown several advantages over other eukaryotic or prokaryotic expression systems: ability of *P. pastoris* to grow at very high cell-densities (with a rapid growth rate) and an almost protein-free extracellular medium facilitating the purification procedures; high levels of foreign proteins expressed under the methanol-induced Promoter AOX1; post-translational modifications like N-glycosylation (although with different oligosaccharide side chain length), methylation, acylation, proteolytic maturation and proper subcellular targeting of the heterologous polypeptide [130].

Furthermore, this yeast expression system is under intensive investigation to eliminate the disadvantages of methanol-oxygen consumption in batch-fed-large scale fermenters, Wang and collaborators engineered a new methanol-free *P. pastoris* by modifying transcription factors regulating AOX1 promoter and developing an efficient glucose-glycerol-shift induction bioprocess for foreign protein expression control. The newly constructed strain could efficiently replace the traditional glycerol-methanol induction in the wild-type strains using an insulin model polypeptide expression, exhibiting a more economic, safe and environment-friendly impact that has a great potential for the biopharmaceutical industry [130]. 

Finally, a crucial parameter that must be considered to achieve high levels of protein expression in yeast expression systems is the use of optimized yeast codon-usage.

For many years it has been empirically assumed that was a relationship between ribosome translation speed and the levels of an exogenous protein synthesized. Codon-usage choice evolved as means to optimize single mRNA translation, essentially by changing the rare codons present in the heterologous gene into those preferred by the expression system (codons found in most highly expressed endogenous proteins). This is a common approach that was also employed for optimal expression of seed Saporin and its derived fusion chimeras in *P. pastoris* [131]. Protein expression in eukaryotic cells is not solely controlled by translation initiation factors. In exponentially growing cells, polypeptide elongation factors (eEF1A, eEF2, and eEF3) may exert powerful translational control [132]. Chu and colleagues recently reported that when rare codons are positioned nearby the start of the polypeptide coding region, translation pausing interferes with making the initiation codon available for loading of the next 40S subunit which may be rate-limiting for initiation and therefore for the overall protein synthesis. Translation efficiency is therefore the result of an interdependence of ribosome association either by de novo initiation or by recycling where both the elongation and initiation factors are contributing to the final protein expression level. For the translation of protein toxins it is of the utmost importance that the polypeptide is efficiently segregated into the ER lumen as the signal peptide emerges from the polysomes. For Saporin expression in Tobacco protoplasts, it was shown that BIP (or PDI) signal peptides may be among the most efficient [124].

Working on codon-usage optimization for *Pichia pastoris* expression Saporin reached just 10 times lower levels of secreted active polypeptides as compared with those of Saporin-KQ, an inactive catalytic mutant [131]. 

Intrinsic host cell toxicity was also consistently decreased, employing this optimization strategy, as also demonstrated for the expression of ATF-Saporin or other chimeric fusions better expressed in *P. pastoris* when a codon-usage optimized ATF domain (human) was used or an optimized single chain variable domain to obtain scFv fusions, such as the anti CD22 scFv termed 4KB that was obtained from the subcloning of VH and VL regions of a parental 4KB128 monoclonal antibody then used to construct Saporin-based ITxs. Target CD22^+^ human Daudi lymphoma cells could internalize this recombinant scFv with absolute immunospecificity as demonstrated by competitive inhibition by the parental monoclonal CD22 antibody [131].

Interestingly, we compared the expression of ITxs made by using two different toxic domains both capable of irreversibly blocking protein translation: PE40 of bacterial origin (endowed with eEF-2 ADP-ribosylation activity) or Saporin. We evaluated optimal microbial expression conditions of various scFv fusion formats made with the two toxin domains to then further evaluate the immunospecific cytotoxic performance of each different fusion construct, together with their achieved yields in *E. coli* or *P. pastoris* expression systems [131]. A number of fusion constructs were designed and expressed either in *E. coli* or in *Pichia pastoris* and the resulting fusion proteins affinity-purified. Protein synthesis inhibition assays showed that the selected recombinant ITxs were active, having comparable IC_50_ (inhibitory concentration by 50%) in the nanomolar range. Overall our results confirmed that *E. coli* is the system of choice for the expression of recombinant fusion toxins of bacterial origin (that otherwise underwent proteolytic degradation in the yeast cells). We demonstrated, in contrast, that Saporin-based ITxs are best expressed and recovered from *P. pastoris* cultures following yeast codon-usage optimization. Codon-optimization of the scFv domain would appear important in order to increase the potential number of secreting clones that were capable of producing at least 1–2 mg/L of fusion protein. In addition, among alternate design options, the best performing ones were those having a yeast codon-optimized scFv where a 18-amino hydrophilic 18-flexible linker GSTSGSGKPGSGEGSTKG, (amino acid one letter code) that showed enhanced resistance to proteolysis together with reduced aggregation of scFvs [132,133] was inserted for joining the VH and VL variable chains which were fused to the N-terminus of mature Saporin sequence through a tri-alanine linker.

The scFv versions having either a (G_4_S)_3_ linker between the VH and VL or other formats tested were either not producing viable clones or gave rise to high yields of mutated Saporin fusions that were found inactive. 

## 12. Conclusions

Saporin-based immunotoxins and chimeric toxins could represent potentially good candidate tools for the treatment of human malignancies, but there are still several hurdles to overcome related to the issues of immunogenicity, endothelial toxicity, intracellular delivery/efficient release and heterologous fusion toxin expression, all of which might be considered as vital to the successful clinical development of Saporin-based therapeutics. 

We have tried to review those that are already promising, suggesting additional possible interventions using genetic engineering approaches that may prove important for their future development as anticancer drugs. 

Furthermore, the possibility to have new recombinant antibodies available as humanized molecules in a relatively short time and cost effective (several companies offer this service, see for example http://www.evitria.com) or to directly select scFv from humanized libraries, together with the possibility to express recombinant RIPs and to eventually engineering their sequences, may allow again for future attractive clinical applications of ITxs. 

## Figures and Tables

**Table 1:** The therapeutic effects of some selected Saporin-based immunotoxins in preclinical in vivo models of human malignancies. ALL, acute lymphoblastic leukemia; B-NHL, B-cell non-Hodgkin’s lymphoma; T-ALL, T-cell acute lymphoblastic leukemia; ALCL, anaplastic large cell lymphoma.

IT/TT	Target Antigen(s)	Preclinical Model	Comment	Reference
BU12-Saporin	CD19	SCID Mouse-NALM-6 (pre-B ALL)	BU12-Saporin treatment gave a significant prolongation in survival	[33]
Ber-H2/Saporin	CD30	SCID mouse-JB6 (ALCL)	Significant inhibition of solid tumor growth in Ber-H2-Saporin treated animals	[40]
HB2-Saporin + OKT10-Saporin combination	CD7 and CD38	SCID-HSB-2 T-ALL	Combination of two ITx significantly better therapeutically	[43]
BU12-Saporin + OKT10-Saporin + 4KB128-Saporin	CD19, CD22 and CD38	SCID mouse-Ramos (B-NHL)	Individual ITx curative of only a proportion of animals. Combination of all three ITs curative of all	[44]
BU12-Saporin + rituximab	CD19 and CD20	SCID mouse-Ramos (B-NHL)	Combination of IT + rituximab antibody significantly better than individual monotherapies	[45]
EV20-SAP	HER3	SCID Mouse-melanoma (cells)	EV20-Saporin treatment significantly reduced pulmonary metastases in a melanoma xenograft model	[46]

**Table 2:** Clinical studies with Saporin-based immunotoxins and bispecific antibodies. PR, partial response; MTD, maximum tolerated dose.

IT/TT	Target Antigen	Disease	No. of Patients	Phase	Comments	Reference
BER-H2/SO6	CD30	Hodgkin’s lymphoma	4	pilot	3 PRs	[40]
BsAb1 + BsAb2	CD22	B-cell lymphoma	5	pilot	4 PRs	[41,42]
BU12-Saporin	CD19	B-cell lymphoma	8	I	MTD not reached	Flavell et al. unpublished results (1996)
OKT10-Saporin	CD38	Myeloma	10	I	MTD not reached	Flavell et al. unpublished results (2001)
BU12-Saporin	CD19	Pediatric ALL	5	I	MTD not reached	Flavell et al. unpublished results (2002)
